# Degradation of Organelles or Specific Organelle Components via Selective Autophagy in Plant Cells

**DOI:** 10.3390/ijms15057624

**Published:** 2014-05-05

**Authors:** Simon Michaeli, Gad Galili

**Affiliations:** Department of Plant Science, the Weizmann Institute of Science, Rehovot 76100, Israel; E-Mail: simonm@weizmann.ac.il

**Keywords:** Arabidopsis, selective-autophagy, autophagosome, ATG8, reticulophagy, chlorophagy, pexophagy, mitophagy, rubisco-containing bodies, plant organelles, vacuole, degradation

## Abstract

Macroautophagy (hereafter referred to as autophagy) is a cellular mechanism dedicated to the degradation and recycling of unnecessary cytosolic components by their removal to the lytic compartment of the cell (the vacuole in plants). Autophagy is generally induced by stresses causing energy deprivation and its operation occurs by special vesicles, termed autophagosomes. Autophagy also operates in a selective manner, recycling specific components, such as organelles, protein aggregates or even specific proteins, and selective autophagy is implicated in both cellular housekeeping and response to stresses. In plants, selective autophagy has recently been shown to degrade mitochondria, plastids and peroxisomes, or organelle components such as the endoplasmic-reticulum (ER) membrane and chloroplast-derived proteins such as Rubisco. This ability places selective-autophagy as a major factor in cellular steady-state maintenance, both under stress and favorable environmental conditions. Here we review the recent advances documented in plants for this cellular process and further discuss its impact on plant physiology.

## 1. Introduction

**Figure 1.**
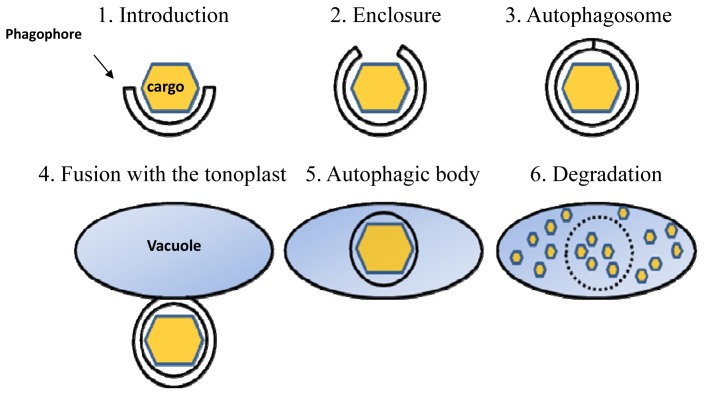
A simplified schematic representation of autophagy. (**1**) A phagophore is generated in the vicinity of the cytosolic cargo; (**2**) Through the action of autophagy-related proteins (ATG), the phagophore elongates to engulf the cargo; (**3**) The full enclosure of the phagophore generates the autophagosome containing the cargo; (**4**) Following its trafficking to the vacuole, the outer membrane of the autophagosome fuses with the tonoplast; (**5**) The cargo, now surrounded by a single membrane (autophagic body), is released into the vacuole lumen; (**6**) The autophagic body is degraded within the vacuole along with its cargo.

Eukaryotic cells accumulate a considerable amount of biological waste during their life cycle, including aged proteins, protein aggregates, malfunctioning ribosomes and damaged organelles. All of this biological waste has to be removed to prevent its possible toxic effect [1] and reused as a recycled energy supply [2,3]. One of the major cellular processes responsible for this is autophagy, which literally means ‘self-eating’, and generally accounts for three distinct clearance pathways: (i) chaperone-mediated autophagy [4], which has so far not been reported in plants; (ii) microautophagy [5,6]; and (iii) macroautophagy. Of these three types, macroautophagy is the best characterized process and will be termed from here onward simply as autophagy. This process involves the sequestration of a cytosolic portion of the cell through its engulfment by an autophagosome, a double-membrane vesicle that originates from a double membrane precursor termed isolation membrane or phagophore [7,8] (Figure 1, steps 1–3). The autophagosome is then directed towards the cell’s lytic compartment (the lysosome in animals and the vacuole in plants and fungi) where its outer membrane fuses with the membrane of the lytic compartment (tonoplast in plants) (Figure 1, step 4). Upon fusion with the tonoplast, the cargo present within the autophagosome, which at this stage is surrounded by a single membrane and termed the autophagic-body, is released into the vacuole lumen for degradation [9,10] (Figure 1, steps 5 and 6). This apparently enables the recycling of the autophagosome cargo for reuse as an energy source.

Nearly 30 genes that control autophagy were originally identified in unicellular fungi, and most are also conserved in animals and plants [9,11]. Some of these conserved autophagy-related genes (abbreviated as *ATG* genes) encode proteins of the core autophagy machinery that are responsible for the entire set of processes presented in Figure 1 [12,13]. Autophagy operates at a basal level under favorable (non-stress) growth conditions [14–17] and is significantly induced following stress. Disruption of single copy *ATG* genes encoding for individual proteins of the core autophagy machinery, e.g., *ATG5* or *ATG7*, resulted in plant hypersensitivity to a variety of stresses [16,18–25].

Autophagy has been originally considered to be a non-selective process, mediating the bulk degradation of cytosolic components in response to acute stresses. Yet, in recent years, autophagy has also been shown to operate as a selective process, termed selective-autophagy. Selective autophagy operates to dispose of specific targets, such as individual proteins, protein aggregates, organelles, and even invading pathogens, by their delivery for degradation in the lytic compartment [9,26,27]. In animals, the processes of the bulk and selective autophagy are currently at the forefront of cellular research, as it seems that autophagy deficiency associates with numerous illnesses [28–30]. In contrast, the study of selective autophagy in plants is still in its infancy. Two examples of plant processes regulated by selective autophagy include disposal of protein aggregates (aggrephagy), mediated by the plant NBR1 cargo receptor [25,31,32], and selective autophagy of specific proteins, such as the RNA silencing component Argonaute1 [12] and the porphyrin scavenger, Tryptophan-rich Sensory Protein (TSPO) [33]. These examples and additional research suggests that selective autophagy also plays an important role in plant growth and response to stress (for a recent review see [26]). Nevertheless, information regarding selective autophagy of plant cells organelles, such as the selective clearance of plant peroxisomes and mitochondria, is only starting to emerge. Here we review the current knowledge regarding selective autophagy of plant intra-cellular organelles or organelle components, and address the physiological significance of these processes.

## 2. ATG8, a Central Protein Mediator of Selective Autophagy

**Figure 2.**
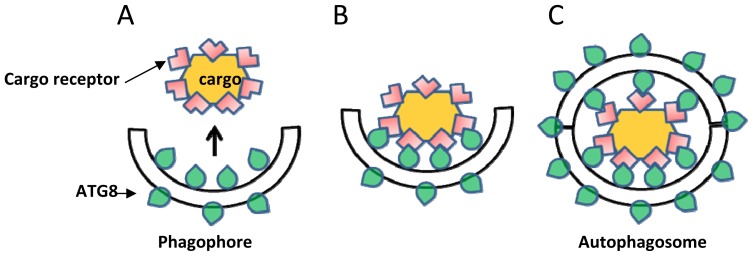
A simplified scheme demonstrating mediation of selective autophagy by ATG8. (**A**) A phagophore is decorated with ATG8 proteins (illustrated as a green drop); (**B**) ATG8 recognizes and binds the cargo receptor (pink L-shape) that is located on the surface of the cargo, resulting in its anchoring to the expanding phagophore; and (**C**) The full enclosure of the double membranes generates an autophagosome that contains the cargo and is decorated with ATG8 proteins.

Autophagy requires the formation of a double membrane vesicle termed the autophagosome; extensive reviews of the entire set of proteins involved in the formation of the autophagosome are available, e.g., in [9,13,34,35], and therefore this issue is not covered here. Among these proteins, ATG8 is one of the central best-studied proteins of the core autophagy machinery [11,36]. ATG8 is involved in the lipidation of the autophagosome precursor membrane (phagophore) by a ubiquitination-like process. This process acts to conjugate phosphatidylethanolamine (PE) to the expanding phagophore membrane until its full enclosure to form the autophagosome (Figure 1). Because ATG8 is continuously attached to both autophagosomes and autophagic-bodies, it is the most widely used protein marker for their detection [37]. In plants, similarly to other organisms, the accumulation of ATG8-labeled autophagosomes is significantly enhanced following biotic and abiotic stresses causing energy deprivation, such as carbon (C) or nitrogen (N) deficiency [19,21–24,38,39]. As a consequence of their role in autophagosome biogenesis, ATG8 proteins are essential for both bulk and selective autophagy. Yet, in selective-autophagy, ATG8 proteins fulfill an additional role in the selection of specific cargo to be sequestered prior to its degradation. ATG8 proteins bind to specific proteins containing an ATG8 Interacting Motif (AIM), which are either themselves targets of selective autophagy, or serve as specialized receptors (or adaptors) that mark the target cytosolic cargo or organelles for degradation [9,26,27,40] (see also Figure 2). This process is sometimes mediated by a ubiquitination event where certain cargo receptors attach to ubiquitinated proteins residing on the surface of degradation-destined organelles [27,40]; in other cases, this process is mediated by specific ATG8-interacting proteins residing on the target organelle. A well-studied example is ATG32, a protein that is localized to the outer membrane of yeast mitochondria. ATG32 binds ATG11 and ATG8 to mediate selective autophagy of mitochondria (mitophagy) in yeast [41,42]. Compared to the single copy gene of ATG8 in yeast (*Sacharomyces cereviseae*), two isoforms exist in Drosophilla and *C. elegans*, seven in humans and nine in the model plant *Arabidopsis thaliana* [11]. The relatively large family of ATG8 proteins in plants apparently implies that ATG8 carries more complex functions in selective-autophagy in plants that in non-plant organisms, probably due to their sessile nature that demands intricate mechanisms to cope with changing environmental conditions.

## 3. Selective-Autophagy of Plant Organelles

### 3.1. Selective Autophagy of ER (Endoplasmic-Reticulum) and ER Components

**Figure 3.**
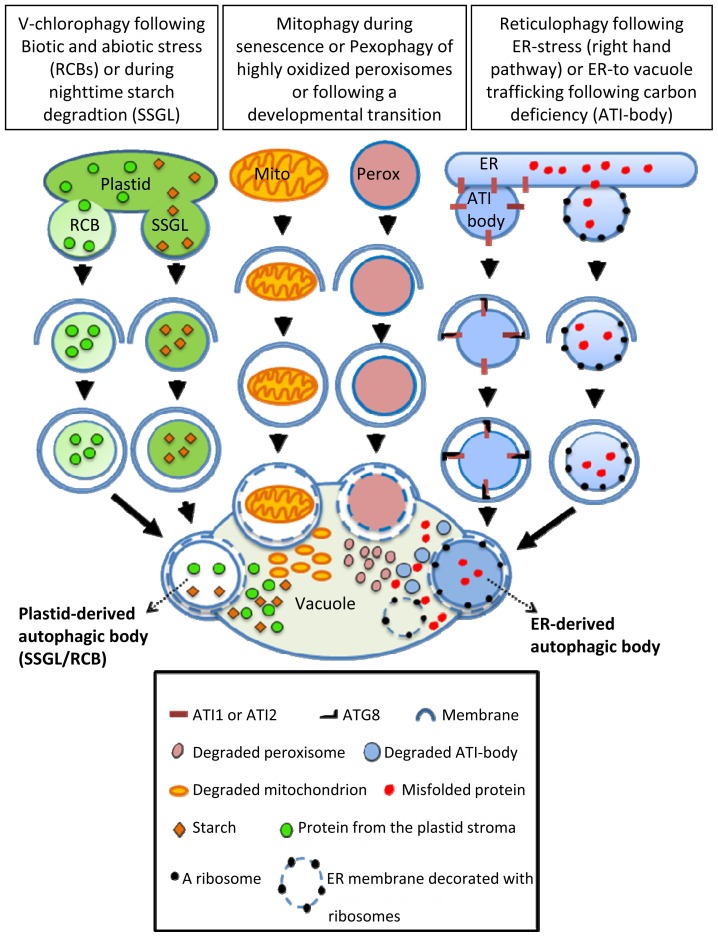
A scheme illustrating all currently known modes of clearance of plant organelles or their components by selective-autophagy. Abbreviations: Mito, mitochondrion; Perox, peroxisome; ATI-body, ATI1 and ATI2 positive bodies; RCB, Rubisco-containing body; SSGL, small starch granule-like vesicle.

In animals and yeast it has been shown that endoplasmic reticulum (ER) components are sequestered into autophagosomes and are then delivered to the vacuole by selective autophagy, a process sometimes termed as reticulophagy [43,44]. This type of selective autophagy is especially induced following stress that triggers the accumulation of malfolded proteins within the ER (a phenomenon known as ER-stress) that stimulates the Unfolded Protein Response (UPR) [45]. ER-stress is also induced through the application of chemicals that disrupt protein folding, such as Tunicamycin and Dithiothreitol (DTT). Indeed, treating Arabidopsis plants with these chemicals induced the mobilization of the ER-resident fluorescent marker GFP-HDEL (GFP fused to the HDEL amino-acid sequence for retention in the ER) to the vacuole by vesicles that co-localize with the autophagosome marker cerulean-ATG8e. Moreover, autophagic-bodies containing ribosome-decorated ER membranes were detected within the vacuole lumen [21]. These data demonstrated the existence of reticulophagy in plant cells (Figure 3). Furthermore, the Arabidopsis ER-stress sensor IRE1b, which is involved in transporting UPR signals from the ER to the nucleus, is required to trigger this reticulophagy [21]. Nonetheless, though reticulophagy is considered as a selective-autophagy process, the autophagy adaptor protein(s) driving this process in plants have not yet been reported.

A new ER-to-vacuole transport route, defined by two closely related Arabidopsis ATG8-binding proteins termed ATI1 and ATI2, was recently identified in Arabidopsis plants [46]. These proteins are specific to plants [46] and apparently bind to more than one ATG8 isoform [47]. A closer examination of ATI1 fused to GFP as a representative revealed that under regular, non-stress growth conditions it is partially associated with the ER membrane, and following dark-induced carbon starvation, it becomes associated with newly formed bodies that move along the ER membrane and are subsequently transported into the vacuole (Figure 3) [46]. Notably, these special bodies (termed ATI-bodies) are distinct from mitochondria, peroxisomes, Golgi and also from “classical” autophagosomes, defining a unique type of stress-induced, autophagy related compartment that is apparently involved in the delivery of ER components to the vacuole [9,46]. Proteins that bind ATG8 usually possess an ATG8 Interacting Motif (AIM), which in animals is also termed Light-chain 3 Interacting Region (LIR) [27,40,48]. Indeed, ATI1 (as a representative) possesses two AIMs located on opposite sides of a predicted trans-membrane domain. The sequence of the *N*-terminal AIM appears closer to the AIM/LIR consensus sequence than that located close to the *C*-terminal region [46,48]. Overexpression or suppression of *ATI1* stimulates or suppresses seed germination on media containing the germination suppression hormone Abscisic-acid (ABA) [49]. Since ATI-bodies are distinct from “classical” autophagosomes, the ATI1 and 2 proteins are apparently not involved in the ER-stress-induced process of reticulophagy. Yet, clarifying this issue requires further research.

Finally, several other studies also discuss the possibility that autophagy is also involved in the direct ER-to-vacule trafficking route of vacuole-resident proteins, such as seed storage proteins, reflecting also a non-degradative role for autophagy (for a review discussing this issue see ref. [50]). Yet, whether ATG8 is involved in this trafficking route is still unknown [50]. Nonetheless, the ATI-body was suggested as a good candidate for transporting vacuole-residing proteins directly from the ER to the vacuole [9,50].

### 3.2. Selective Autophagy of Plastids and Plastid Components

Some plastid proteins were shown to be degraded via the ubiquitin-proteasome system (UPS) [51] as well as by the action of plastid endogenous proteases [52]. Yet, as plant plastids hold a considerable amount of the C and N leaf pool, it might prove energetically advantageous for plant cells to degrade and reuse not only specific plastid components, but also entire plastids. Indeed, the delivery of un-developed plastids as well as senescing plastids in individually darkened leaves (IDLs) to the vacuole via an autophagy-like process has been reported [53,54]. The absence of plastid-to-vacuoles trafficking in the autophagy deficient *atg4a4b* double-mutant directly supports the involvement of the core autophagy machinery in this process [54]. Furthermore, several reports demonstrated the involvement of selective autophagy in the turnover of some individual plastid components without the dismantling of the entire photosynthetic apparatus. A seminal report [55] describes a special type of Rubisco Containing Body (RCB) observed both in the cytoplasm and vacuoles of naturally senescing wheat leaf cells [55]. Ultra-structure observations further revealed the occasional engulfment of the RCB by a multi-layer membrane, suggestive of autophagy. Indeed, the transport of RCBs to the vacuole requires active autophagy as this process does not occur in an Arabidopsis autophagy-deficient *atg5* mutant exposed to darkness [56]. In addition, RCBs labeled by a chloroplast-targeted DsRed fluorescent protein co-localized with the GFP-ATG8 autophagosome marker, confirming that autophagy is involved in the delivery of RCBs to the vacuole [56] (Figure 3). The induction of plastid-derived vesicles was also reported to occur following avirulent *Pst* DC3000 (*AvrRps4*) infection, leading the authors to speculate that RCBs might be an important source for reactive oxygen species (ROS) or other signaling molecules that induce plant defense response [57]. Exposure to salt stress also triggered the induction of RCBs in rice plants, where RCBs were formed in chloroplast protrusions (CPs), a known feature of stressed plastids [58]. Vesicles containing Rubisco were also reported by a different group that named them Senescing-Associated Vesicles (SAVs) [59,60]. These SAVs are acidic compartments that contain SAG12, a senescence associated protease, and a membrane localized vacuolar H^+^-ATPase [59,60]. Thus, the SAV may represent a small type of vacuole that executes degradation that is probably distinct from RCBs. Moreover, active autophagy is probably not involved in SAV biogenesis, as SAVs were also detected in the background of the *atg7* autophagy deficient mutant [60].

Interestingly, RCBs are not the only plastid-derived bodies whose biogenesis and function requires active autophagy. The autophagy-dependent disposal of plastid starch has also been recently reported. In this research [17], increased autophagic activity during the night was observed in tobacco plants grown under favorable conditions. Additionally, following treatment with a chemical inhibitor of autophagy and following silencing of several ATG genes, inhibition of starch degradation was detected. The involvement of autophagy in starch degradation was finally confirmed by detecting the double-labeling of both CFP-ATG8a (autophagosome marker) and Granule-bound Starch Synthase I fused to YFP (GBSSI-YFP) in small spherical structures, termed Small Starch Granule-Like (SSGL) structures [17] (Figure 3). An interesting conclusion drawn from this work is that autophagy is also involved in an important energetic function during the night, which is not necessarily associated with stress. This notion is also strengthened by the fact that autophagy-deficient mutants display reduced growth under short-day photoperiod [15]. Additionally, the phenotype of a starchless and *atg* double mutant showed a more severe growth retardation as well as earlier cell death, compared to either a mutant in a single ATG gene or in a starchless mutant [15]. A major distinction between bulk and selective-autophagy is that bulk-autophagy is generally induced during relatively severe or extended stresses, while selective autophagy operates also under favorable growth conditions or following mild stresses [9]. Thus, the delivery of starch via SSGLs under non-stress conditions is apparently operated by selective autophagy. Also, the degradation of plastid components via RCBs seems to be operating via selective autophagy as it is specifically linked to leaf carbon status but not to the nitrogen status [61]. Furthermore, the amount of RCBs generated in the background of starchless mutants is higher than their amount in starch excess mutants [61]. Thus it is probable that RCBs and SSGLs are distinct bodies although this still awaits experimental evidence. Plastid stromal protein degradation was shown to occur, but being incomplete in *atg7* and *atg5* mutants, indicates that active autophagy is not absolutely required for plastid stromal protein degradation [62]. This suggests that other degradation pathways, possibly proteases and the UPS, act in concert with autophagy to degrade plastid components.

On the basis of the annotation of selective autophagy of other organelles (such as mitophagy for mitochondria), several reports termed the process of whole chloroplast targeting to vacuole as chlorophagy [57,62,63]. We thus propose the term “Vesicular Chlorophagy” (or V-Chlorophagy) to be used for distinguishing between chlorophagy and the selective removal of specific vesicles containing plastid components, such as Rubisco or starch. “Vesicular Chlorophagy” represents the manner by which chloroplast derived vesicles are apparently being identified by the autophagy machinery and are delivered to the vacuole. Notably, to the best of our knowledge, this type of selective autophagy, where the degradation of specific components of an organelle rather than the entire organelle are targeted for degradation, is apparently unique to plants and has so far not been reported in any other species.

### 3.3. Selective Autophagy of Peroxisomes (Pexophagy)

Peroxisomes are compartments that are important for proper plant metabolism, development and response to the environment [64]. This organelle requires controlled protein import, as well as tight control over its proteins turnover. In the case of the later, it is especially important in the transition from β-oxidation of fatty acids in the germinating seedling to its role in photorespiration in later developmental stages. Indeed, during this developmental transition, the glyoxylate cycle enzymes, isocitrate lyase (ICL) and malate synthase (MLS) are degraded [64,65] possibly via the UPS [65,66]. Other possible candidates that can play a role in matrix protein degradation are peroxisomal proteases, though none of these were shown to degrade ICL or MLS directly [67]. In an effort to learn more about the function of the peroxisome-specific protease LON2, Farmer and colleagues used a forward genetic approach to identify novel genetic interactions with *LON2* [68]. The known resistance of *lon2* mutants to indole-3-butyric acid (IBA)-induced lateral root formation was used in a screen for suppression mutants of this phenotype. Remarkably, the three different suppressor mutations identified in the screen were all in autophagy-related genes, namely, *ATG7*, *ATG2* and *ATG3*. This suppression was clearly assigned to peroxisomal defects as the morphology of *lon2* peroxisomes (larger and less abundant than in WT) was restored following autophagy disruption [68]. Apparently, peroxisomes lacking a functional LON2 protein are recognized through the autophagic machinery as targets for degradation via a pexophagy pathway. Unfortunately, a description of the possible physiological implication of the double *lon2-atg* mutants was not presented in this study. It is also unclear whether the apparently larger peroxisomes in *lon2* mutants are the result of increased size or of the aggregation of regularly sized peroxisomes. In another study, a peroxisomal fluorescent marker was utilized in a screen aimed at identifying mutations that affect peroxisomal cellular positioning (PEroxisome Unusual Positioning (*PEUP*) mutants). Remarkably, ATG2 and ATG7 were also identified in this screen, as well as ATG18a. [69]. Peroxisome degradation was inhibited in the *peup/atg* mutants, and they contained aggregated peroxisomes that were highly oxidized. Peroxisome aggregation was induced by application of hydrogen peroxide, and peroxisome aggregates were shown to be co-localized with an ATG8 marker [69]. Taken together these results suggest that peroxisomes that are damaged by hydrogen peroxide aggregate and are then selectively degraded by the autophagy machinery (Figure 3). This study was conducted mainly on mesophyll protoplasts isolated from leaves of three weeks old plants. Notably, the quality control of plant peroxisomes is probably tissue-dependent as it appears to be more pronounced in leaves than in roots [70]. As mentioned earlier, peroxisomal proteins turnover is also vital during the developmental transition that enables seedling establishment. The involvement of autophagy in peroxisomal proteins turnover during this developmental stage was demonstrated in a study that showed impaired peroxisome degradation in the hypocotyls of Arabidopsis *atg5* and *atg7* mutants during seedling growth (Figure 3). Interestingly, also here pexophagy activity was organ-dependent as expression of *ATG7* was higher in hypocotyls than in seedling cotyledons [71]. The manner by which the selectivity of plant pexophagy is determined, *i.e.*, the possible function of pexophagy-specialized cargo receptors, is currently unknown. Nevertheless, the fact that plant pexophagy is specifically targeting damaged peroxisomes, strongly suggests that it operates selectively [70]. Recently, electron-loose structures were visualized by electron microscopy of autophagy mutants and were stained with anti-ATG8a antibodies. These structures, probably representing a precursor of a phagophore, were located specifically in the vicinity of abnormal peroxisomes, providing further evidence to support the selectivity of plant pexophagy [70].

The molecular mechanism of pexophagy is relatively well documented in yeast cells. In *Pichia pastoris* ATG30 is anchored to peroxisome membrane by its interaction with two types of peroxins (PEX14 and PEX3), where it acts in pexophagy by recruiting ATG11 and ATG17 [72]. The PEX3 protein of *S. cerevisiae* is the docking site of ATG36, a cargo receptor for pexophagy that interacts with ATG11 and probably indirectly with ATG8 [73]. In mammals, the NBR1 cargo receptor that interacts with ATG8, and that was classically assigned to the autophagy-dependent degradation of protein aggregates (aggrephagy), was recently found to be essential for pexophagy [74]. ATG30 and ATG36 seem to be restricted to fungi since homologs were not found in other organisms [72,73]. On the other hand, a homolog of NBR1 does exist in plants [31,32] and was shown to act in plant aggrephagy [25]. Thus it will be interesting to evaluate the possible involvement of plant NBR1 in pexophagy. Taken together, it is now clear that, similarly as in yeast and mammals, pexophagy also operates in plants. Nevertheless, the identity of the proteins required for this process in plants is yet to be determined.

### 3.4. Selective Autophagy of Mitochondria (Mitophagy)

The degradation rates of various mitochondrial proteins are highly variable [75] suggesting the operation of several independent degradation processes, such as mitochondrial proteases, the UPS and mitophagy [75]. Although very well established in yeast and animals [76], evidence supporting mitophagy in plants is limited [77]. A recent report identified a plant ATG11 protein (whose sequence also contains ATG17 related domains) that is apparently fundamental for autophagosome delivery to the vacuole, but is not essential for autophagosome biogenesis [78]. Similarly to other essential *atg* deficient plants, *atg11* mutants display premature senescence and increased sensitivity to N and C limitations. Fungal ATG11 proteins were implicated in selective mitophagy, pexophagy and the Cytoplasm to Vacuole Targeting (CVT) pathway [35]. This encouraged the authors to examine the possible involvement of ATG11 in Arabidopsis mitophagy. Indeed, following dark-induced senescence, mitochondrial proteins, as well as mitochondrion numbers, remained stable in the background of *atg7-2* and *atg11-1* mutants, compared to WT plants. Additionally, a mitochondrial marker, visible in the vacuoles of wild type plants following senescence, was not detected in the vacuoles of these autophagy mutants. Finally, co-localization of the mitochondrial marker with both ATG8a and ATG11, confirmed their involvement in the clearance of plant mitochondria from the cytoplasm to the vacuole [78]. The cellular mechanisms that regulate the specificity of plant mitophagy still await discovery. It will also be very interesting to look at the determinants that target a specific mitochondrion for disposal and the proteins that are involved in this process.

## 4. Conclusions and Future Perspectives

In recent years we have witnessed a tremendous leap in experimental evidence for selective autophagy in plants, as well as in our understanding of the context in which it operates. These studies show that selective autophagy is a central regulator of the response of plant cells, mainly to environmental stresses. Nevertheless, major gaps still exist in our knowledge of the machinery involved in these processes, mainly, the identification of novel ATG8-binding proteins as well as deeper elucidation of the involvement of ubiquitination processes. In addition, open questions still remain regarding the physiological significance of chlorophagy, V-chlorophagy, reticulophagy and pexophagy in plant growth and response to environmental cues, particularly stresses. In addition, the identification of novel stress-induced components that are involved in these processes may contribute to enhance plant stress tolerance. Judging from the relatively high rate in which this field has gained progress in recent years, we believe that answers to these questions will soon be revealed.
